# Development of Antibacterial Thermoplastic Starch with Natural Oils and Extracts: Structural, Mechanical and Thermal Properties

**DOI:** 10.3390/polym16020180

**Published:** 2024-01-08

**Authors:** Jorge Luis López Terán, Elvia Victoria Cabrera Maldonado, Judith del Carmen Araque Rangel, José Poveda Otazo, María Isabel Beltrán Rico

**Affiliations:** 1 Grupo de Investigación de Moléculas y Materiales Funcionales (MoléMater), Facultad de Ingeniería Química, Universidad Central del Ecuador, Ritter s/n y Bolivia, Quito E. C. 170521, Ecuador; jllopez@uce.edu.ec (J.L.L.T.); evcabreram@uce.edu.ec (E.V.C.M.); 2 Grupo de Investigación de Moléculas y Materiales Funcionales (MoléMater), Facultad de Ingeniería en Geología, Minas, Petróleos y Ambiental, Universidad Central del Ecuador, Jerónimo Leyton y Av. La Gasca, Quito C. P. 170521, Ecuador; juditharaque@gmail.com; 3 Departamento de Ingeniería Química, Universidad de Alicante, Apdo. 99, 03080 Alicante, Spain; jpo25@alu.ua.es

**Keywords:** thermoplastic starch, biopolymer, biodegradable, essential oils, antimicrobial activity

## Abstract

In this study, the influence of the incorporation of eucalyptus (EO), tea tree (TT) and rosemary (RO) essential oils and Chiriyuyo extract (CE) on the structure and properties of thermoplastic starch (TPS) obtained from potato starch, glycerin and water was evaluated. All oils and the extract were used at a concentration of 0.5 g/100 g of TPS, while for TT, the effect of the concentration was also studied. The mixtures obtained were processed by extrusion and thermocompression molding. The sheets were characterized by XRD, FTIR, TGA, SEM and analyses of their mechanical properties, antimicrobial characteristics and biodegradability. The results show that the use of small concentrations of the oils in 70TPS does not induce changes in the TPS structure according to the results of XRD, FTIR and TGA, with each essential oil and CE affecting the mechanical properties unevenly, although in all cases, antimicrobial activity was obtained, and the biodegradability of TPS in soil was not modified. An increase in the concentration of TT in 60TPS causes marked changes in the crystallinity of TPS, providing a greater modulus with a higher concentration of TT. Regardless of the amount of TT, all sheets maintain antimicrobial characteristics, and their biodegradation in soil is delayed with a higher oil content.

## 1. Introduction

Currently, global policies are orienting plastics industry strategies toward the synthesis of products obtained from renewable resources [1], especially from underutilized biomass, in order to reduce the environmental pollution derived from the use of non-biodegradable polymers [2,3]. For example, the European Union Directive 2019/904, which entered into force in June 2019, refers to the impact of single-use plastic products and requires a significant and sustained quantitative reduction in the consumption of these products by 2026 [4]. Bio-based polymers of natural origin, extracted from plants, cereals and invertebrate animals or synthesized by microorganisms, are the main choice for sustainable materials due to their biocompatibility, biodegradability and lack of toxicity, which provides excellent potential for investigating industrial applications. To date, cellulose, starch, chitosan and polylactic acid (PLA) are the natural polymers most used to obtain bioplastics [5,6,7,8]. Unfortunately, most bioplastics have higher processing costs and worse mechanical and thermal properties than conventional plastics, with their main advantage being the ability to biodegrade in an adequate period of time. Bioplastics also generate a significant decrease in the carbon footprint and contribute to the circular economies of countries [7].

Starch is the most abundant natural polysaccharide and is biodegradable, easy to handle and low-cost. Its use as a bioplastic is generating great interest in the scientific community. Starch is mainly made up of two polymers: amylose and amylopectin. The main chains of both are formed by glucose ring monomers (glucopyranose), including numerous short branches in the case of amylopectin. The rigidity of the monomers compared to the main chains of most conventional thermoplastics in frequent use, much simpler and non-polar, together with the high molecular weight of both amylose and amylopectin and the crystallinity of the latter, provides starch with high rigidity. Therefore, starch must be dissolved or plasticized to be processed. The most common technique for processing starch is casting, in which the starch is dissolved in water (conc of 4–8% *w*/*w*). After evaporating it, thin films with reasonably good properties are obtained. The casting technique, however, suffers from the fact that it requires evaporating a large amount of solvent (water) and that it is limited to obtaining films of small thickness. Starch can also be processed like conventional thermoplastics in the presence of plasticizers (usually combinations of water and glycerol or other alcohols) through the application of shear and heat (usually by extrusion or injection molding); the starch is gelatinized, melts and is moldable. This starch is known as thermoplastic starch (TPS). TPS has the advantages that it can be molded into the form of thin films or sheets of greater thickness or into other more complex forms and that the process and reagents are economical, and, of course, it is biodegradable [8,9].

However, the difficulty in processing and plasticizing such complex molecules implies that starch bioplastics present worse mechanical properties than petroleum-based plastics, and their properties are dependent on moisture and can decompose during processing if appropriate measures are not taken [9].

In the few known industrial applications of starch as a biopolymer, it is usually combined with other polymers, either synthetic non-biodegradable or biodegradable polymers (PE, PP, poly-caprolactone polyester, polyamide, poly (butyleneadipate-co-terephthalate), rubbers, polyvinyl alcohol, etc.). Although starch applications as bio-based plastics are still scarce, there are several start-ups and larger companies like BASF working to produce bioplastic packaging that could replace traditional plastics. Zeomic™ and Microgarde™ are examples of antimicrobial films developed as active packaging materials. TIPA^®^ manufactures compostable films for the food packaging industry [10,11]. These films are manufactured with existing machinery and are between 20 and 80% bio-based and 100% compostable; however, these films are highly dependent on the environmental humidity, which promotes the degradation mechanisms of the material. Nevertheless, the production of starch bioplastics based on 100% natural products with properties similar to those of petroleum-based plastics is still a challenge for the scientific community [12].

There are a large number of scientific publications aiming to improve the properties of starch films obtained by casting by adding different plasticizers and other additives, such as nanoclays, essential oils, fibers, etc. TPS bioplastics have received less attention. The extracts obtained from the leaves and stems of plants have had excellent results in the development of various types of pharmaceutical and cosmetic products and are being used as active additives for plastics. Many studies have proved that essential oils posse antibacterial, antifungal and even antiviral activities [13,14]. Although the mechanisms of the antimicrobial action of essential oils are not clearly identified, it may be mainly due to the presence of phenolic compounds and their derivatives, which are able to act against a wide spectrum of pathogenic bacteria [13,14,15,16,17,18,19]. In the case of TT, it may be related to the hydrophobic nature of various types of compounds present in the oil, mainly the presence of terpinene-4-ol, γ-terpinene and α-terpinene, which can cause irregularities in the structure and the permeability of the membrane or enzymatic systems, which give rise to the leakage of ions and other compounds [18]. Essential oils have become an ecological, innovative and sustainable alternative as additives, especially in the food industry sector [19].

Among the essential oils used as additives in the food, pharmaceutical and, more recently, plastics industries are eucalyptus essential oil (EO) derived from Eucalyptus globulus, which is a species of plant belonging to the Myrtaceae family [20]; tea tree oil (TT), which is obtained through the distillation of the leaves of plants of the genus Melaleuca [21,22,23]; and rosemary essential oil (RO) obtained from a plant belonging to the Lamiaceae family [15]. On the other hand, the plant known as Chiriyuyo in Ecuador is a plant species belonging to the family Crassulaceae (species *Kalanchoe pinnata*, native to Africa and Madagascar). It has been found that the extracts obtained from Chiriyuyo (CE) leaves and stems have good antimicrobial activity against bacteria, fungi and viruses [19,24,25,26,27].

Different publications have been found in which essential oils and extracts were incorporated into starch formulations. These are generally starch films obtained by casting and, in some cases, are edible [19,21,21,28,29]. To a lesser extent, some are TPS sheets. Only two papers have been found where essential oils were incorporated into TPS, as in the case of the work of Azevedo et al. (2019) [15], which included whey protein in TPS, along with montmorillonite and rosemary oil. Valencia-Sullca et al. (2018) [29] used coconut and cinnamon essential oil in chitosan sheets that were joined to cassava TPS sheets by thermocompression in order to provide them with antimicrobial properties.

In this work, TPS sheets processed by extrusion and thermoforming containing potato starch and the essential oils and extract referred to above (EO, TT, RO and CE) were obtained, and the effect of the TT concentration on TPS was also studied. The objectives were to determine whether these oils induce changes in the structure of the material, how they affect its biodegradability and mechanical properties, whether they impart antimicrobial characteristics, and what the most appropriate concentration is that provides antimicrobial characteristics without being a detriment to other properties.

## 2. Materials and Methods

### 2.1. Materials

Potato starch (20.5% amylose and 79.5% amylopectin) was acquired from Finnamyl Ld (Kokemäki, Finlandia). Glycerol was purchased from Labbox Labware S.L. (Vilassar de Dalt, España). Zinc stearate was obtained from Across Organics (Geel, Belgica). Eucalyptus essential oil (EO), tea tree essential oil (TT) and rosemary essential oil (RO) were provided by Munay Care, Casa del Químico Ecuador and Corquimia Industrial S.L., respectively. Chiriyuyo extract (CE) was obtained by supercritical CO_2_ extraction of Kalanchoe Pinnata leaves at the Chemical Engineering Faculty of the Central University of Ecuador. In the following, we refer to EO, TT and RO as “the essential oils” and to CE as the extract. The main components of EO, TT, RO and CE obtained by GC-MS (Agilent Technologies 7820A/Columna HP-5ms Ultra Inert, NIST 2014 Library), according to the bibliography [30], are presented in Appendix A.

### 2.2. Sheet Composition

In the first series of experiments, the effects of the different types of oils were studied. In this series, a paste containing 70% starch, 18% glycerol and 12% water (70TPS) was prepared. The starch was first mixed with glycerol and stirred thoroughly, and then distilled water was added and mixed to obtain a uniform paste. As a thermal stabilizer, 1 g of zinc stearate per 100 g of the paste was added. To the previous paste, 0.5 g of EO, TT, RO or CE/100 g of TPS was added. Table 1 shows the composition and nomenclature of these samples.

In the second series, the effect of the amount of essential oil was addressed. TT was selected, given the good set of properties demonstrated. To improve the flexibility of the samples, this study was conducted on mixtures containing 60 g starch/100 g TPS, keeping the glycerol/water ratio at 3/2. The composition and nomenclature of the samples prepared in this second series are also shown in Table 1.

### 2.3. Sheet Preparation by Extrusion and Compression Molding

**Figure 1:**
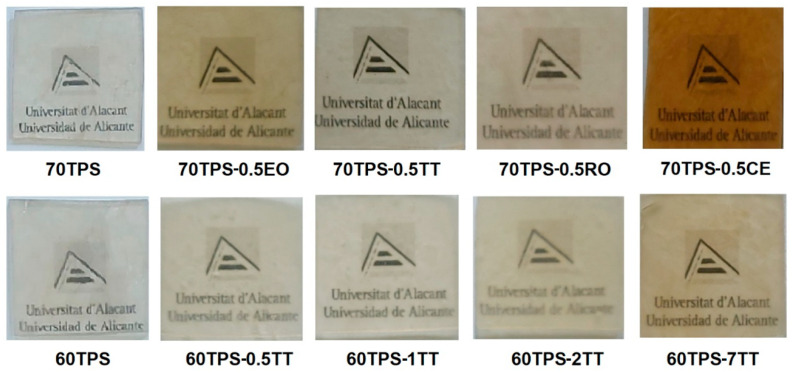
Aspects of the TPS sheets.

The extrusion of the different mixtures was carried out using a single screw extruder (Brabender Plasticorder with the data-processing unit PL-2000) with a screw diameter of 25 mm, length of 500 mm and cylindrical die of 2 mm. The screw speed (80 rpm) and temperature profile (110 °C, from the feeder to the die) were selected to obtain a completely gelatinized material [31]. After extrusion, thermoplastic threads were used to prepare sheets by thermocompression using a thermostated hydraulic press. A piece of thread (~40 g) was placed between Kevlar sheets on a stainless-steel cylindrical mold to obtain sheets with a 16 cm diameter and 1 mm thickness. The plate’s temperature was set to 152 °C for 18 min. For the first 4 min, the press was closed gradually and opened twice to remove the water vapor, thus avoiding bubbles in the sheets. Then, the pressure was increased up to 8 MPa and maintained for 14 min. Finally, a cold plate press at 16 °C was used to cool down the sheets. The resultant sheets were stored for at least one week at 23 °C and a relative humidity of 56% before testing. Figure 1 shows the aspects of the 1 mm thick sheets.

All sheets have a good appearance and certain turbidity. The 60 and 70TPS sheets are colorless, while those containing oils have a certain yellowish or brown coloration (for the CE), which, in all cases, coincides with the color of the oil or extract studied.

### 2.4. Starch and Sheet Characterization

#### 2.4.1. XRD

The crystal structure was studied by XRD. Diffractograms were obtained with a Bruker X-ray diffractometer (D8-Advance model, Ettlingen, Germany). This equipment has a KRISTALLOFLEX K 76080F X-ray generator (power = 3000 W, voltage = 20–60 kV, intensity = 5–80 mA) that has an X-ray tube with a copper anode. The dimensions of specimens were 1 × 1 cm^2^, and the equipment was operated at 40 kV and 40 mA, with 2θ varying from 4 to 50° with a step size of 0.05°.

#### 2.4.2. Fourier Transform Infrared Spectroscopy (FTIR)

The absorption spectra in the infrared region were obtained by Fourier Transform using the spectrophotometer JASCO FTIR 4700 equipped with a germanium-encapsulated KBr beam splitter in the region between 4000 and 500 cm^−1^ at a resolution of 4 cm^−1^.

#### 2.4.3. Thermogravimetric Analysis (TGA)

The TGA of starch and TPS sheets was performed with a Perkin Elmer Pyris TGA 7 equipped with Pyris software. The temperature was changed from 30 to 600 °C at 10 °C/min under a nitrogen flow of 60 mL/min.

#### 2.4.4. Scanning Electron Microscopy (SEM)

The microstructural analysis of starch and the sheets was carried out using the SEM technique with a JEOL JSM-840 electron microscope. The sample sheets first underwent cryofracture by immersion in liquid nitrogen. Images were captured using a 10 kV acceleration voltage. Liquid nitrogen was used to achieve a brittle fracture surface.

#### 2.4.5. Mechanical Properties

An INSTRON 4411 dynamometer was used to measure the maximum tensile strength (TS), elongation at break (E) and Young’s modulus (YM). The tests were carried out on 8 Halterio-type specimens punched out from each sheet. The mechanical test of the specimens was carried out according to the ASTM D882-12 (2012) standard [32], with a jaw separation speed of 50 mm/min.

#### 2.4.6. Antimicrobial Activity Using the Disk Diffusion Method

The bacterial strains used to evaluate the antimicrobial activity were (a) Gram-positive bacteria, *Staphylococcus aureus* ATCC 25923, and (b) Gram-negative bacteria, *Pseudomonas aeruginosa* ATCC 27853. The bacterial strains were obtained from the American Type Culture Collection. The positive controls were Ceftazidime 30 µg Oxoid^®^, Erythromycin 15 µg Oxoid^®^ and Oxacillin 5 µg Oxoid^®^ (Sigma-Aldrich, St. Louis, MO, USA), and the negative control was deionized water.

The antibacterial activity of the sheets was tested using the agar disk method [33]. Müeller–Hinton agar was obtained from Himedia^®^. All tests were performed in triplicate. The sheets were cut into 6 mm punch size holes by using a paper puncher under sterile conditions. The bacterial inocula were prepared in sterile saline solution (0.85% *w*/*v* NaCl) from a fresh culture of each bacterial strain replicated on Müeller–Hinton agar until obtaining turbidity corresponding to the pattern of McFarland No. 0.5 (1.5 × 108 CFU/mL) (Hardy Diagnostics^®^) for each of the bacterial strains. Next, the inoculum of each microorganism was seeded on the agar surface with a sterile swab, and the biopolymer disks, water (negative control) and reference antibiotics for each microorganism (positive control) were placed on the inoculated agar. For incubation, the Petri dishes were placed in an oven at 37 °C for 48 h. Finally, the readings of the results were taken by measuring the inhibition halos at 48 h and expressing the diameter of the inhibition zone in millimeters (mm). The inhibition halos of each biopolymer were compared with the inhibition halos of the positive controls, and the test was considered negative when microbial growth was observed around the disks.

#### 2.4.7. Biodegradability in Vegetable Compost

The determination of the degree of disintegration was carried out by means of an adaptation of the ISO 20200:2004 standard [34]. Synthetic compost was prepared (50% water, 40% sawdust, 30% rabbit food, 10% mature compost, 5% sucrose, 4% corn oil and 1% urea). Specimens of 10 mm × 10 mm (15 specimens of each formulation) were cut from the sheets and introduced into polypropylene meshes. The specimens were introduced and conveniently separated in a polypropylene reactor with dimensions of 30 cm × 20 cm × 10 cm, together with 1 kg of compost. On the sides of the reactor and on the cover, 6 holes of 5 mm diameter were drilled to allow air circulation. The reactors were placed in an oven at 58 ± 2 °C (Memmert SNB 400 and J.P. Selecta S.A., Barcelona, Spain) for 40 days, ensuring a thermophilic incubation period. To ensure the correct composting process, it was necessary to maintain the water level specified by the ISO standard in the reactors. On days 10, 20, 30 and 40 from the start of the test, 3 specimens were extracted from each formulation. After extraction, the specimens were cleaned to remove compost from the surface and dried in an oven at 40 °C to constant weight. The degree of disintegration was calculated by normalizing the sample weight of the specimens with respect to their initial weight [35,36,37].

## 3. Results and Discussion

### 3.1. XRD

**Figure 2:**
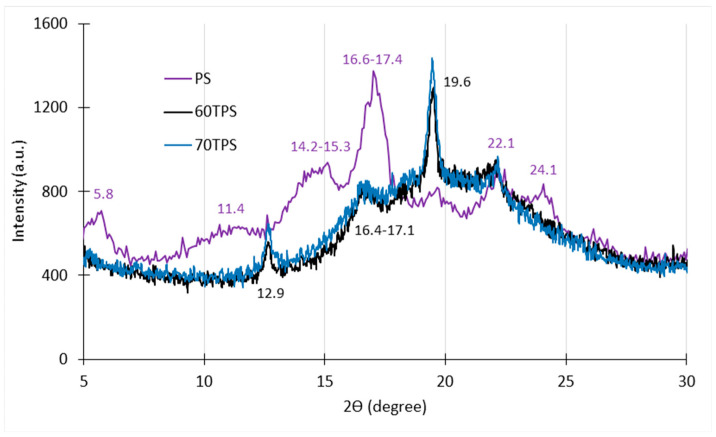
XRD diffractograms of starch (PS), 60TPS and 70TPS.

The diffractograms of starch (PS), together with those of the 60 and 70TPS samples, are shown in Figure 2. Potato starch presents B-type crystallinity, which is typical of starches obtained from tubers, with diffraction peaks at 2θ angles of 5.8° and 11.4°, two unresolved doublets between 14.2°–15.3° and 16.6°–17.4°, and three smoother peaks at 19.7°, 22.1° and 24.1°, which is in accordance with the results presented by other authors for potato starches [38,39].

In the 70TPS sample, some of the characteristic peaks of the B-type structure of potato starch disappear, including those at 5.8° and 11.4°, the doublet at 14.5°–15.2° and the peak at 24.1°, which would indicate that, as a consequence of the effect of the shear and temperature applied during the processing of TPS, the destruction of the starch granules occurs, with the original crystallinity of the starch disappearing to a large extent (but not completely). The small peaks of virgin starch at 22.1° and 19.7° are maintained, and in particular, the latter becomes the peak with the greatest intensity in the 70TPS diffractogram. Moreover, in 70TPS, the starch doublet between 16.6° and 17.4° resolves toward lower angles. The presence of peaks regarding the original structure of starch indicates that, after the thermocompression process, a completely plasticized amorphous material is not obtained, and the appearance of new peaks indicates that, during cooling and aging, restructuring occurs with the formation of new crystals. The positions of two new peaks (12.9° and 19.6°) indicate the formation of V_H_-type crystals. Altayan et al. 2021 [39] prepared TPS sheets obtained by extrusion from wheat and corn starch. They also found that the incomplete destruction of the starch granules, an important ordered recrystallized fraction and a smaller amorphous fraction were obtained, which, despite the differences in the peak positions, which are dependent on the starch origin, are completely consistent with the results obtained in this work. No remarkable differences were obtained between 60TPS and 70TPS.

Most of the results found in the recent literature are about starch films obtained by casting (dissolution and evaporation of the solvent). They show much simpler diffractograms than those shown here for TPS [38,40,41], with an unresolved wide peak between 17 and 21°, indicating the important contribution of the new amorphous phase formed in the casting films. Domene et al. (2019) [38] deconvoluted this wide peak, and their results indicated that some remaining A-type or B-type crystals from the original starch were present (A or B type depending on the starch’s botanical origin), while a new V-type crystalline structure was formed. A comparison between the XRD patterns of the starch casting films obtained by those authors and the TPS sheets (extruded and compressed) obtained in this work, as well as those reported by Altayan et al. (2021) [39], indicates that the disruption of the original starch structure would be greater in casting, as would the presence of the amorphous phase. Anyway, as mentioned previously, TPS processing is much more versatile and economical than casting.

The diffractograms of 70TPS sheets including 0.5 g/100 g of the essential oils (shown as Supplementary Material) have similar patterns to that of 70TPS, probably due to the small amount of oil used. It is worth noting the greater relative intensity that the extract (CE) causes in the peaks at 12.9° and 19.6°, which would indicate a greater extension of V_H_ crystals in the sheets containing the extract.

**Figure 3:**
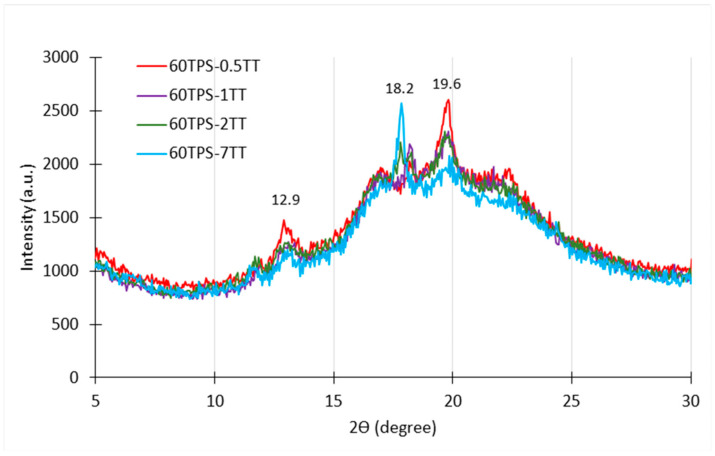
XRD diffractograms of the 60TPS thermoplastic starch and formulations with 0.5, 1, 2 and 7 phr of TT.

Finally, Figure 3 shows the effect of the TT concentration on 60TPS. It is very striking that when increasing the amount of TT oil from 0.5 to 7 g/100 g of TPS, there is a marked change in the crystal structure of the sample; the characteristic peaks of TPS at 12.9° and 19.6° gradually disappear, while new peaks appear at 18.2° (in 60TPS-0.5TT, 60TPS-1TT and 60TPS-2TT) and at 17.8° (in 60TPS-7TT). The higher the oil concentration, the higher the peak intensity. This peak, according to the literature, is associated with the formation of V_A_ crystals, which take place with materials with lower moisture content than V_H_ crystals [39]. As discussed below, the lipid nature of essential oils helps in reducing the affinity of hydrophilic polymers for water, which could help to explain the formation of V_A_-type crystals in TPS at a higher TT concentration.

### 3.2. FTIR Spectroscopy

**Figure 4:**
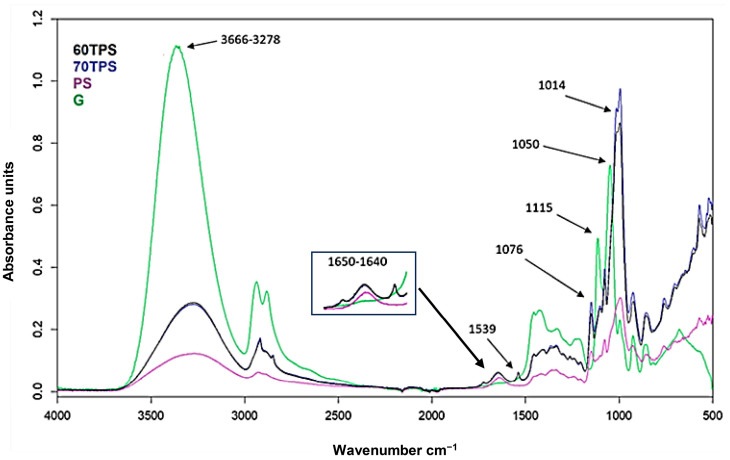
FTIR spectra of PS, glycerol (G), 60TPS and 70TPS.

Figure 4 shows the FTIR spectrum of the main components, starch and glycerol, together with those of 60 and 70 TPS. Due to the similar functional groups of the main components, most of the absorption bands overlap in the TPS spectra, making the interpretation difficult. The broad hydroxyl vibration bands at 3000 and 3600 cm^−1^ (located at 3278 cm^−1^ and 3366 cm^−1^ for starch and glycerin, respectively) are shifted to 3294 cm^−1^ and 3298 cm^−1^ for 60TPS and 70TPS, respectively. In TPS, as a result of the applied shear forces and the temperature reached during the extrusion process, part of the crystalline fraction of starch disappears, as shown in Figure 2, allowing the hydrogen bonds between the hydroxyl groups of starch to be replaced by new hydrogen bonds between starch and glycerol.

The typical band of water molecules adsorbed by the amorphous region of starch granules is found at 1645 cm^−1^ [42,43,44], and it presents a high intensity and is shifted to 1650 cm^−1^ in 60TPS and 70TPS.

In both TPS samples, a small band at 1539 cm^−1^, which comes from the ZnSt used as a thermal stabilizer, can also be observed. Moreover, a small band at 1725 cm^−1^ can be seen, indicating the presence of carbonyl groups. This band is somewhat more intense in 70TPS, where it is accompanied by a small band at 1263 cm^−1^, indicating the formation of ester groups. The combination of both bands could indicate that there has been slight crosslinking between the starch and glycerin molecules or that the partial degradation of TPS has taken place. Either of the two could be a consequence of the effect of shear and temperature, which would be more intense in 70TPS, since it possesses more viscosity due to its lower content of the plasticizer, and consequently, this sample suffers a more intense shear during processing. In any case, the extension of this process must be very small, given the low intensity of the band, and these samples have no color.

In the region between 1500 and 800 cm^−1^, the starch has a wide band at 994 cm^−1^ originating from the stretching vibration of C-O in the C-O-C groups of glucose and two bands of lower intensity at 1150 and 1077 cm^−1^, characteristic of the C-O stretching vibrations of C-O-H groups. These C-O-H bands appear at 1115 and 1050 cm^−1^ in glycerin. The spectrum of TPS reflects the presence of the bands of both components but with an important shift in the mentioned glycerin bands, which move to lower wavenumbers, which reflect the destruction of hydrogen bonds between glycerin molecules as they are replaced by new glycerin and starch hydrogen bonds.

The FTIR absorption spectra of the essential oils have the expected characteristic CH stretching bands between 2900 and 2300 cm^−1^. The broad OH stretching band at 3000–3600 cm^−1^ is also present in the oils and is relatively very intense for CE due to its high hydroxyl group content as a consequence of the extraction process. All of them, except for TT, present a C=O stretching band between 1700 and 1740 cm^−1^, which is in accordance with the results presented by other authors, as well as the C-O stretching of terpenoid components between 1000 and 1120 cm^−1^. On the other hand, monoterpene vibrational modes, characteristic of essential oils, are observed at wavenumbers of 886, 1436 and 1644 cm^−1^ [25,45,46]. Due to the small amounts of essential oils included in 70TPS, there are no remarkable changes in the FTIR spectrum of 70TPS containing oils (Figure 4). Very small bands between 1716 and 1724 cm^−1^, together with a band at 1251 cm^−1^, corresponding to the presence of carbonyl groups and C-O bonds, are observed due to the active compounds present in these natural products, such as carboxylic acids, ketones, aldehydes or esters in sheets containing RO, CE and EO [46]. However, TT does not possess carboxyl groups in its composition; therefore, that band in the 70TPS-0.5TT sample must come from the partial oxidation of hydroxyl groups into carbonyl groups, which may indicate the chemical crosslinking or partial degradation of these sheets.

**Figure 5:**
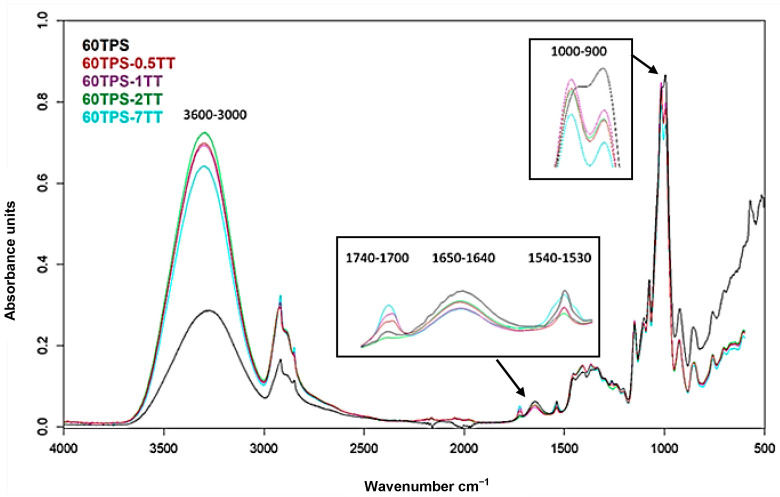
FTIR spectra of TPS sheets and 60TPS formulations with 0.5, 1, 2 and 7 phr of TT.

The FTIR spectra of the 60TPS sheets containing different amounts of TT show no significant differences (Figure 5). It is only worth noting the progressive shift in the tension band of the hydroxyl group, which moves from 3291 cm^−1^ in 60TPS to 3305 cm^−1^ in 60TPS-7TT, and the lower intensity of the band at 1650 cm^−1^, indicating lower water adsorption when the oil is included.

### 3.3. Thermogravimetric Analysis

**Figure 6:**
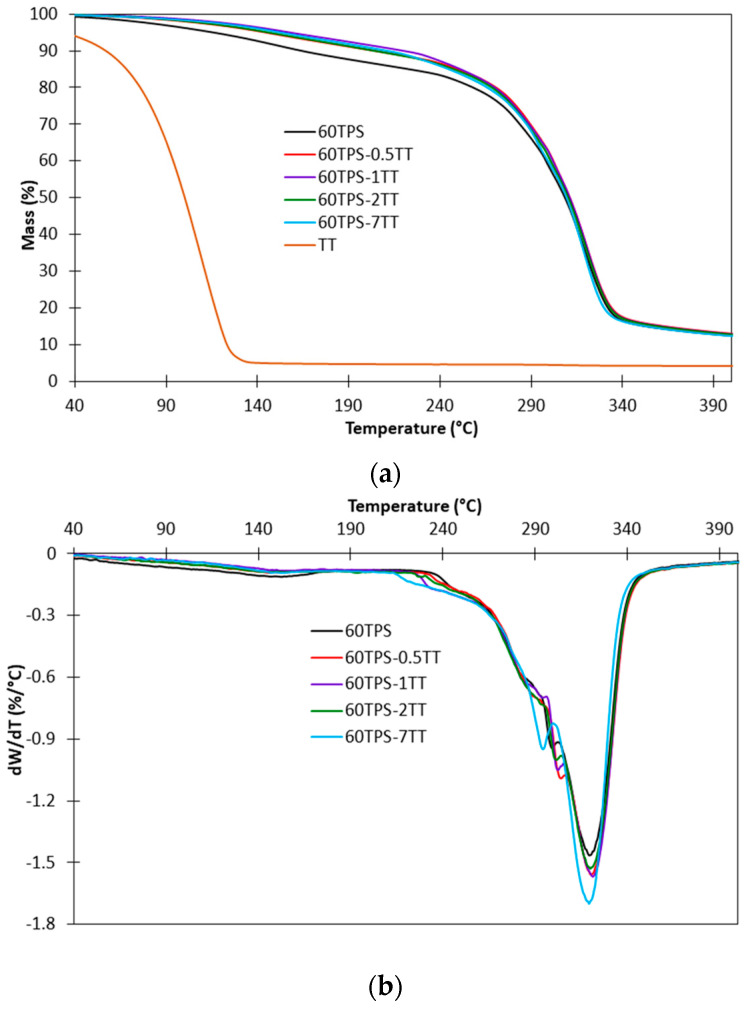
TG (**a**) and DTG (**b**) curves of 60TPS with different amounts of TT.

Figure 6 shows the TG and DTG curves of TT, 60TPS and 60TPS sheets with different TT contents. The TG and DTG curves of 70TPS samples containing different types of oil are not shown since the small amounts of oils included in the 70TPS series are barely reflected in the TG curves, as happens for 60TPS-0.5TT, as shown in Figure 6.

The volatile components of the oils, as well as water and glycerin, evaporate at relatively low temperatures. For example, in the TG experimental conditions (a dry nitrogen flow), TT (Figure 6) presents the maximum decomposition rate (Tmax) at 110.1 °C, with a residue of 4.3% at 380 °C, while Tmax for water is 85 °C and for glycerin is 190 °C, and neither presents residues (water and glycerin TG curves have not been included for clarity). Therefore, it is expected that any residues of these components that have not interacted adequately with the TPS paste evaporate while the sheets are processed by the extrusion and thermoforming processes.

60TPS decomposes in two distinct stages. Under the test conditions, the first stage takes place between 40 and 190 °C and is due to the gradual evaporation of the remaining water and glycerin, which have weaker interactions with starch [47,48]. It is striking that in all TPS sheets containing TT, the first stage of decomposition presents less weight loss than the 60TPS sheets. In fact, the weight loss for 60TPS sheets is 12.4% at 190 °C, while for sheets containing TT, it ranges between 8.8 and 7.6%. According to these results, TT provides a reduction in the moisture content of the sheets regardless of the concentration, which is consistent with the reduction in the intensity of the band at 1650 cm^−1^ observed in FTIR. Due to their lipid nature, essential oils help reduce the water vapor permeability of hydrophilic polymers [15,49,50], affecting the water retention capacity of the sheets. This decrease in moisture content values can improve the stability of the sheets and prevent premature degradation, which generates greater durability.

During the second and main stage of the decomposition of TPS, several overlapping processes take place. The main one consists of the decomposition of amylose and amylopectin in starch, which, when found alone, presents a symmetrical DTG peak around 321 °C [48,49,50]. In the case of the 60TPS sheet shown in Figure 6b, it is interesting to highlight the area that precedes Tmax between 220 and 310 °C. Two shoulders and a small peak are observed around 300 °C. These shoulders and the peak are dependent on the type of starch, the plasticizer concentration and the heating rate (results to be published) and have been attributed to a fraction of the starch that, given its structure, suffers major interactions and is strongly plasticized by water and glycerin. In the case of sheets containing TT, this peak shifts to lower temperatures with increasing TT concentration (304.6, 301.6, 300.6 and 294.2 °C for TT concentrations of 0.5, 1, 2 and 7 g/100 g TPS), which may be related to the changes in the crystalline fraction of starch that have been observed in XRD for the peaks at 2θ of 12.9° and 19.6° and to the appearance of the new peak at 18.2° (Figure 3).

A reduction in the crystallinity of the material would allow major interactions among the starch, the plasticizers and the TT. In any case, this is indicative of the important modifications that the increasing content of oil introduces in the TPS, despite the fact that a part of it may have evaporated during the processing of the sheets. The residues at 600 °C are very similar for all samples and are in the range of 13.4 to 13.9%.

### 3.4. Scanning Electronic Microscopy

**Figure 7:**
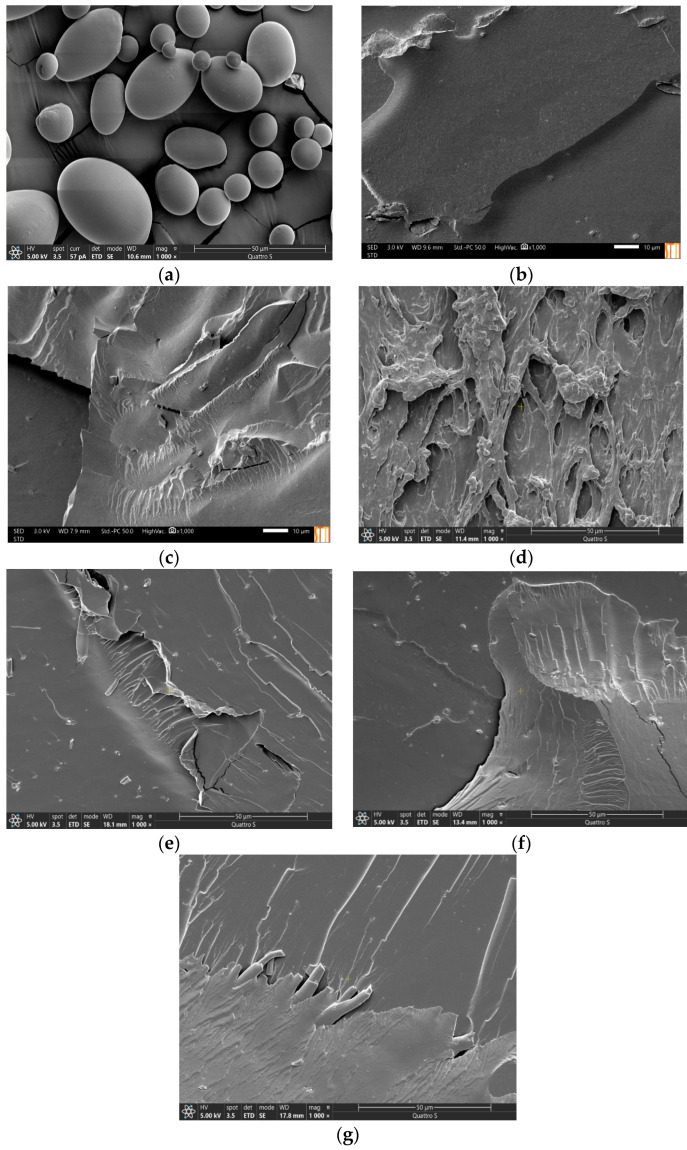
SEM micrographs of fracture surface: PS (**a**), 60TPS (**b**) and 70TPS (**c**) containing 0.5% EO (**d**), TT (**e**), RO (**f**) and CE (**g**) at 1000× magnification.

To know in greater detail the structures of the biopolymers, micrographs of the cryogenic fracture surface were obtained. The presence or absence of irregularities in the fracture zone could help to interpret the mechanical behavior of the materials. Figure 7 shows the micrographs of the starch granules and those of 60 and 70TPS sheets obtained at 1000× magnification, as well as those of 70TPS sheets with the oils.

The 60TPS sheets (Figure 7b) show a homogeneous surface due to the important destructuring and gelatinization of the starch during the extrusion process, as shown by XRD. It is also possible to appreciate a small fraction of fragments of starch granules that, although they have been broken and lost their entity during extrusion (see Figure 7a), have not been fully integrated with the rest of the material. In the case of the 70TPS sheet (Figure 7c), the presence of small cracks and a fracture surface containing more edges is appreciated, which may indicate greater rigidity, corresponding to a less plasticized system [51,52]. However, the few starch grain fragments observed in 70TPS are much smaller than in 60TPS, probably due to the greater shear applied in the processing of this material as a result of its lower plasticizer content and thus its higher viscosity, as commented above.

Despite the good appearance of the 70TPS-0.5EO sheet (Figure 1), micrographs taken in different parts of the material (Figure 7d as an example) reveal a very irregular fracture surface, which suggests an excessive increase in the viscosity of the extruded material that prevents a good flow to fill the mold. The appearance of the sheets containing TT, RO and CE (Figure 7e–g) is much more similar to that of 70TPS, with a fracture surface that has fewer edges and cracks than 70TPS, where it is also possible to appreciate small fragments of starch grains that have not been incorporated into TPS.

**Figure 8:**
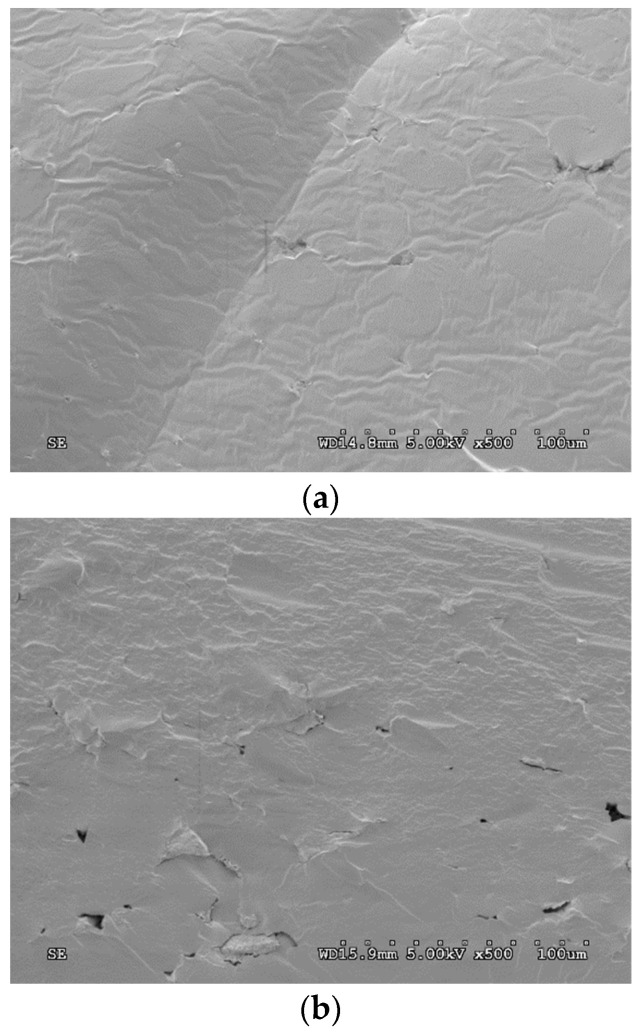

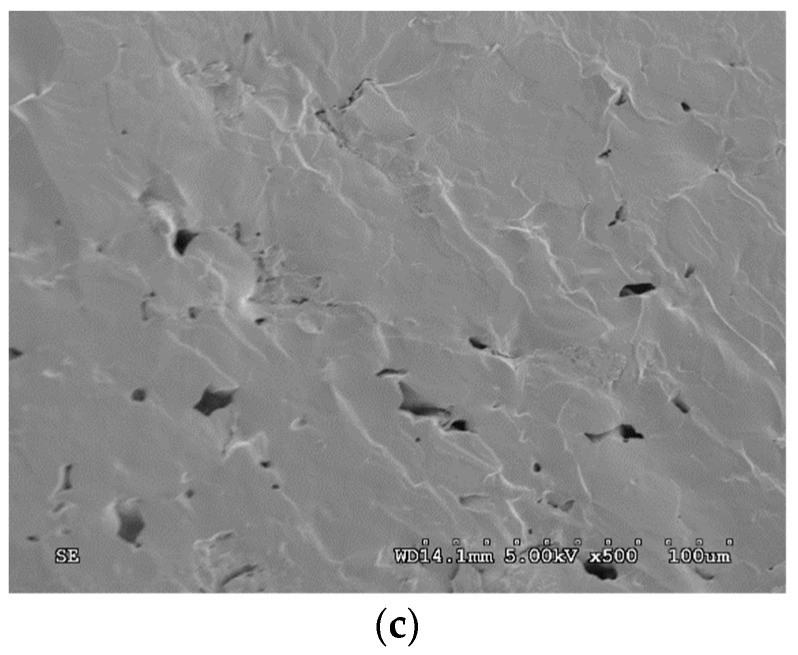
SEM micrographs of the fracture surfaces of (**a**) 60TPS-1TT, (**b**) 60TPS-2TT and (**c**) 60TPS-7TT at 500× magnification.

Figure 8 shows 60TPS-1TT, 60TPS-2TT and 60TPS-7TT sheets at 500× magnification. The incorporation of a small amount of TT (Figure 8b) provides a homogeneous surface with smooth angles, without the appearance of granules, corresponding to the surface of a soft plasticized material. In 60TPS-1TT (Figure 8a), small hollows or cavities are seen in some areas that become larger as the oil content increases, while in the sample 60TPS-7TT, large cavities are appreciated, probably caused by the evaporation or volatilization of the excess TT during processing that could not interact with the starch. Other authors also described similar hollows in starch films prepared by casting that included essential oils [21,53], as did Azevedo et al. (2019) [15] in TPS containing RO. 

### 3.5. Mechanical Properties

Table 2 shows the mean values of Young’s modulus, tension and deformation at break for the 70TPS sheets with different oil types and 60TPS sheets with varying amounts of TT.

As expected, the concentration of the plasticizer plays an important role in TPS, and it is observed that the higher the content of the plasticizer (60TPS compared to 70TPS), the lower the value of the modulus and the stress at break. In the plasticized system, the small plasticizer molecules retained between the huge polymer chains increase the free volume and, consequently, the mobility between the polymer chains [51]. An increase in the deformation at break would also be expected with the increase in plasticizer content, but the 60TPS and 70TPS samples present very close values, taking into account the standard deviations.

The incorporation of EO, TT, RO and CE leads to very significant changes in mechanical properties. These additives, added in small concentrations (0.5 g/100 g of TPS) to the 70TPS sheets, cause an increase in their deformation capacity and tensile strength, which may be related to the crosslinking or decomposition observed by FTIR, which, although to a small extent, could modify the mechanical behavior of the materials. Specifically, EO, with the tangled irregular structure shown in SEM analysis (Figure 7d), causes an important increase in Young’s modulus (70TPS = 28.4 ± 0.5 versus 70TPS-0.5EO = 95.9 ± 3.0 MPa).

Nevertheless, the modulus is substantially reduced in the 60TPS sheets with the incorporation of a small amount of TT, and it increases progressively with the amount of oil, which may be influenced by the changes in the polymer crystallinity observed by XRD. Contrarily, the deformation at break is highly increased by the presence of the oil (60TPS = 53.5 ± 6.7 versus 60TPS-2TT = 226.1 ± 17.8 %), except for the 60TPS-7TT sheet, possibly due to the presence of the large imperfections mentioned in the SEM analysis.

The effect of the inclusion of oil in starch casting films and TPS sheets described in the literature varies substantially between different authors. Jamróz et al. (2018) [49] found that when increasing the concentration of TT in starch films obtained by casting, the tensile strength and modulus were progressively reduced, while the elongation at break increased. Similarly, Gutierrez et al. (2018) found a decrease in Young’s modulus and tension at break in TPS films with the inclusion of 4% w/w blueberry extract [54]. In the case of TPS, Azevedo et al. (2019) [15] found that the addition of RO in small amounts to TPS formulations that included milk protein and Mt decreased the mechanical properties (tensile strength, elongation and modulus).

### 3.6. Antimicrobial Assay

Table 3 shows the results of the antimicrobial activity assays of the materials studied against bacteria, namely, Gram-positive Staphylococcus aureus ATCC 25923 and Gram-negative Pseudomonas aeruginosa ATCC 27853.

The 60TPS and 70TPS samples do not show antibacterial activity against the studied bacteria, but when the essential oils and the Chiriyuyo extract are included, inhibition halos can be observed in all the samples.

Despite the high temperatures and shear to which sheets were subjected during processing, all sheets containing essential oils present antibacterial activity, even with low contents, proving that no denaturation of the oil takes place during processing, despite the fact that the partial evaporation of the oil could take place during processing [53]. All of these sheets, except for those containing EO, show higher activity against the Gram-negative bacteria *Pseudomonas aeruginosa*, which is considered more resistant than Gram-positive bacteria since its outer membrane restricts the diffusion of hydrophobic compounds through its lipopolysaccharide coating.

There are no significant differences in the content of TT, probably due to the higher evaporation of the oil as its concentration in TPS increases. In any case, the kinetics of saturation by the substrate is typical of the enzymatic activity related to the antimicrobial effect observed for the action of various organic compounds [29,53]. The best sheet is 60TPS-1TT, which presents inhibition halos of 15.7 mm for the *Staphylococcus aureus* bacteria and 20.3 mm for *Pseudomonas aeruginosa*. No results showing the antimicrobial activity of TPS containing essential oils have been found in the literature. Silveira et al. (2020) [21] studied cassava starch casting films incorporating TT and found that a concentration of 0.08% TT inhibits the bacteria *S. aureus* and *C. albicans*. These authors proposed that this antimicrobial activity of TT is due to the terpinen-4-ol and 1,8-cineole contained in this oil.

### 3.7. Biodegradability in Compost

**Figure 9:**
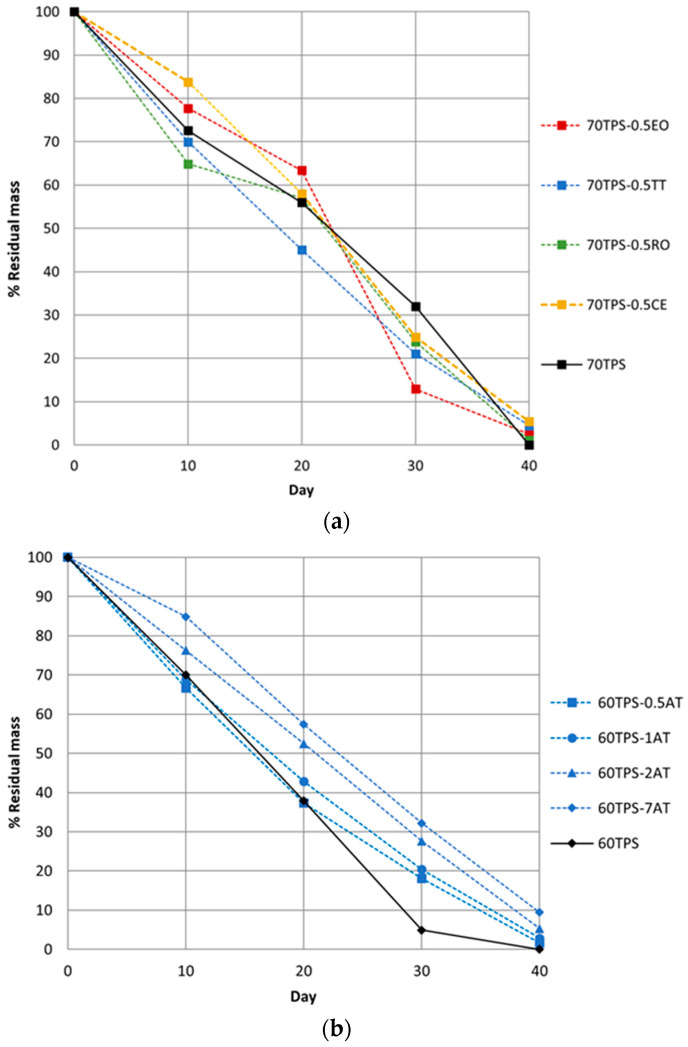
Disintegration degree under controlled compost conditions as a function of incubation time: (**a**) 70TPS sheets with EO, TT, RO and CE; (**b**) 60TPS sheets with different amounts of TT.

The samples were composted in order to check whether the inclusion of the oils modifies the rate of decomposition of the samples in soil. Figure 9 shows the residual percentage weight of all samples subjected to composting. A gradual reduction in the weight of the sheets over time can be seen for all samples. 60TPS and 70TPS present no residue after 40 days of the assay, and 60TPS was probably completely disintegrated days before the end of the trial. 70 TPS sheets containing the different oils present a residual mass after 40 days, which ranges between 5.5 and 1.0% in the order CE > TT > EO > RO (Figure 9a). The delay in the process of biodegradation in soil may be due, on the one hand, to the antimicrobial activity conferred by the oils and, on the other hand, to the lower moisture affinity of the sheets, as commented on previously [36,37,41].

The effect of TT content on 60TPS samples is shown in Figure 9b. As the oil content increases in the 60TPS sheets, a slower biodegradation process is obtained; that is, the higher the amounts of oils, the higher the resistance to degradation in compost. On day 40, all 60TPS sheets containing TT have a residual mass ranging from 1.6% (60TPS-0.5TT) to 9.4% (60TPS-7TT).

## 4. Conclusions

TPS sheets from potato starch including EO, TT, RO and CE as natural additives and different concentrations of TT have been obtained. These additives, when included in 70TPS at a concentration of 0.5 g/100 g of TPS, induce few changes in the structure of the sheets, judging from the results of XRD, TGA and FTIR. In all cases, they confer antimicrobial characteristics against the Gram+ and Gram− bacteria studied while hardly affecting biodegradability in soil. SEM micrographs reveal a good microstructure for all additives except EO, which has a tangent structure. This material is precisely the one that produces a greater increase in the Young’s modulus of the sheets. The other oils cause an increase in both the modulus and stress at break and maximum deformation in 70TPS.

There is a marked effect of the amount of TT used (between 0.5 and 7 g/100 g of 60TPS) on the crystallinity of TPS, which, despite being a plasticized material with strong interactions between starch and plasticizer, presents a crystalline fraction with V_H_-type crystals that disappear with the concentration of oil to produce V_A_-type crystals. Increases in tensile stress at break and maximum deformation are observed with the increasing concentration of TT oil, as well as a delay in the process of biodegradation in soil, which could be related to the changes in crystallinity observed. Nevertheless, judging from the results of TGA and SEM, TT can only be adsorbed by TPS up to a certain extent, after which it evaporates, leaving gaps inside of and on the surface of the samples. The antimicrobial activity is independent of the amount of oil incorporated, which contributes to the saturating effect of TT. According to FTIR and TG results, all samples containing TT have lower humidity than the reference TPS, which can be very interesting from the point of view of its applicability and durability.

## Figures and Tables

**Table 1:** Composition of the studied mixtures. All mixtures contained 1 g of ZnSt per 100 g of TPS.

Nomenclature	Starch (g)	Glycerol (g)	Water (g)	Essential Oil/Extract (g)
70TPS	70	18	12	--
70TPS-0.5EO	70	18	12	0.5
70TPS-0.5TT	70	18	12	0.5
70TPS-0.5RO	70	18	12	0.5
70TPS-0.5CE	70	18	12	0.5
60TPS	60	24	16	--
60TPS-0.5TT	60	24	16	0.5
60TPS-1TT	60	24	16	1
60TPS-2TT	60	24	16	2
60TPS-7TT	60	24	16	7

**Table 2:** Mechanical properties of TPS sheets containing EO, TT, RO and CE.

TPS Sheets	Young’s Modulus (MPa)	Tension at Break (MPa)	Deformation at Break (%)
60TPS	16.5 ± 1.5	2.5 ± 0.5	53.5 ± 6.7
70TPS	28.4 ± 0.5	3.5 ± 0.4	68.7 ± 5.8
70TPS-0.5EO	95.9 ± 3.0	7.3 ± 0.3	106.1 ± 6.8
70TPS-0.5TT	34.8 ± 4.7	5.0 ± 0.3	96.7 ± 9.0
70TPS-0.5RO	47.6 ± 2.8	6.7 ± 0.2	98.2 ± 7.8
70TPS-0.5CE	35.6 ± 3.5	6.0 ± 0.4	136.2 ± 13.1
60TPS-0.5TT	3.0 ± 0.3	2.6 ± 0.1	202.6 ± 16.6
60TPS-1TT	4.6 ± 0.7	2.7 ± 0.2	218.1 ± 13.9
60TPS-2TT	9.5 ± 1.2	3.9 ± 0.3	226.1 ± 17.8
60TPS-P 7TT	14.0 ± 1.2	4.0 ± 0.4	152.7 ± 8.5

**Table 3:** Antimicrobial inhibition halos of TPS sheets containing the oils and extract.

TPS Sheets	Inhibition Zone *S. aureus* (mm)	Inhibition Zone *P. aeruginosa* (mm)
70TPS	--	--
70TPS-0.5EO	14.3 ± 0.7	6.3 ± 0.9
70TPS-0.5TT	12.2 ± 0.8	18.1 ± 0.7
70TPS-0.5RO	11.7 ± 2.4	12.5 ± 4.3
70TPS-0.5CE	11.5 ± 0.8	19.3 ± 1.2
60TPS	--	--
60TPS-0.5TT	15.0 ± 1.9	17.8 ± 0.6
60TPS-1TT	15.7 ± 3.2	20.3 ± 1.9
60TPS-2TT	14.3 ± 2.3	11.0 ± 1.1
60TPS-7TT	15.3 ± 7.5	19.5 ± 4.1
**Positive control**		
Eritromicina (15 µg)	22.0 ± 7.0	--
Oxacilina (5 µg)	33.0 ± 7.0	--
Ceftazidime (30 µg)	---	35.0 ± 7.0

## Data Availability

Data are contained within the article.

## Supplementary Materials

The following supporting information can be downloaded at: https://www.mdpi.com/article/10.3390/polym16020180/s1, Figure S1: Figure S1: XRD diffractograms of 70TPS containing EO, TT, RO and CE.

## Appendix A

**Table A1:** Identification by GC/MS of essential oils.

Active Components	Characterization by GC/MS
*Eucalyptus globulus Labill.* (EO)	Cineol or eucalyptol, monoterpenes: α-pinene, *p*-cymene, limonene; aldehydes: butyraldehyde, capronaldehyde. Azulene, tannins, resin, flavone: eucalyptin; triterpenes derived from ursolic acid.
*Melaleuca alternifoliae.* (TT)	Terpinene-4-ol, γ-terpine, α-terpine, 1,8-cineole, α-pinene, *p*-cymene, terpinolene, α-terpineol.
*Rosmarinus officinalis.* (RO)	α-Pinene, β-pinene, camphene and terpenic esters such as 1,8-cineole, camphor, linalool, verbinol, terpineol, carnosol, rosmanol, isorosmanol, 3-octanone, isobanyl-acetate and β-caryophyllene; vanillic, caffeic, chlorogenic, rosmarinic, carnosic, ursolic, oleanolic, butylinic, betulinic, betulin, α-amyrin, β-amyrin, borneol and bornyl acetate.
*Kalanchoe pinnata.* Chiriyuyo extract (CE)	Hexadecanoic acid, Phytol, 9,12,15-Octadecatrienoic acid, Eicosanoic acid, Diisoctylphthalate, Tetracosanal, Heptacosane, Squalene, Hexacosanal, δ-Tocopherol, Vitamin E, (*E*)-24-Propylidenecholesterol, β-Amyrin acetate, γ-Sitosterol.
