# Supplementary figures and images for: Development of Antibacterial Thermoplastic Starch with Natural Oils and Extracts: Structural, Mechanical and Thermal Properties
Source: Polymers (Basel). 2024 Jan 8;16(2):180. doi: 10.3390/polym16020180 (PMC10818525; doi:10.3390/polym16020180)

## Supplementary Materials

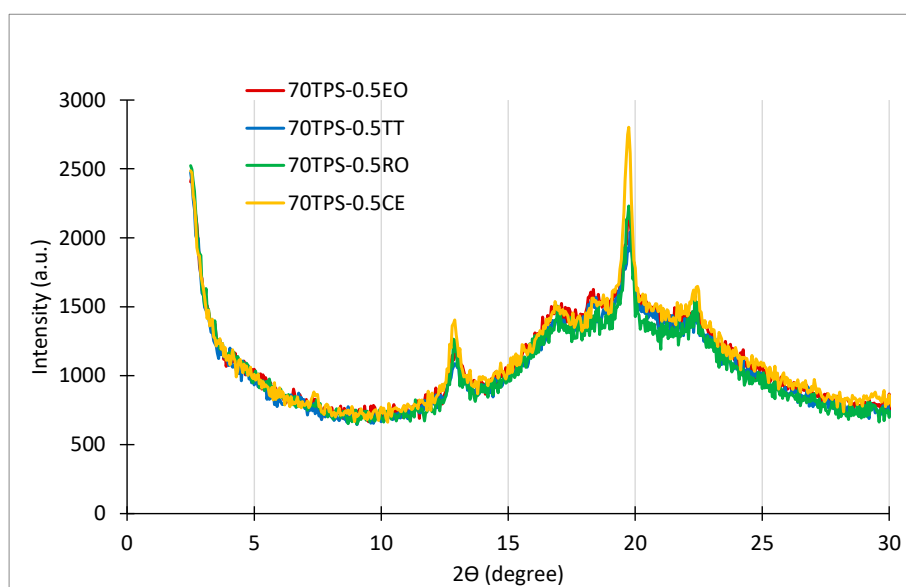

Figure S1: XRD diffractograms of 70TPS containing EO, TT, RO and CE.

Supplement: Supplementary file 1 [file polymers-16-00180-s001.zip › polymers-2796298-supplementary.pdf]
